# Cecal gastrointestinal stromal tumor causing ileocolic intussusception in an adult: A rare case report

**DOI:** 10.1016/j.ijscr.2021.106097

**Published:** 2021-06-08

**Authors:** Bibek Man Shrestha, Suraj Shrestha, Sanjeev Kharel, Shankar Adhikari, Sansar Babu Tiwari, Bishnu Prasad Kandel, Paleswan Joshi Lakhey

**Affiliations:** a Maharajgunj Medical Campus, Institute of Medicine, Kathmandu, Nepal; b Department of GI and General Surgery, Tribhuvan University Teaching Hospital, Institute of Medicine, Kathmandu, Nepal; c Department of Pathology, Tribhuvan University Teaching Hospital, Institute of Medicine, Kathmandu, Nepal

**Keywords:** Gastrointestinal stromal tumor, Ileo-colic intussusception, Bowel obstruction

## Abstract

**Introduction and importance:**

Cecal gastrointestinal stromal tumors (GIST) constitute a rarer subtype of all GISTs. Rarely, it can present with ileocolic intussusception in adults making it a challenging diagnosis due to non-specific clinical features.

**Case presentation:**

A 30-year previously healthy woman presented with lower abdominal pain and a distended abdomen who was subsequently diagnosed with ileocolic intussusception on a CT scan. Intraoperatively, a pedunculated polypoid hard mass was identified in the cecum and thus, a standard right hemicolectomy was performed with the suspicion of malignancy. Histopathology of the resected mass confirmed CD117 negative, spindle type GIST.

**Discussion:**

Cecal GIST presenting in the form of ileocolic intussusception is rare. Contrast-enhanced CT scan is the preferred imaging modality for the evaluation of patients with suspected GIST to determine the extent of the tumor, the presence or absence of metastatic disease alongside evaluation of the possibility of complete resection. Adjuvant imatinib therapy following complete resection decreases the disease recurrence.

**Conclusion:**

Intussusception in an adult can be the first manifestation of underlying malignancy like GIST. Complete surgical resection of the tumor with a negative margin offers long-term survival.

## Highlights

• Ileocolic intussusception is rare in adult and often associated with pathological lead points like neoplasm.
• Cecal GIST is an uncommon cause of ileocolic intussusception in adults.
• Diagnosis of this entity is challenging because of its non-specific presenting features.

## 1 Introduction

Intussusception is an emergency condition where there is a telescoping of one segment of the intestine into an adjoining segment causing gut strangulation and then finally gangrene and perforation. Intussusception in adults comprises 1% of all intestinal obstructions and only 0.003%–0.02% among all hospitalized patients [1]. As high as 65% of adults presenting with intussusception has pathological lead points as neoplasms like polyps, submucosal lipomas, and tumor masses including gastrointestinal stromal tumor (GIST) [2]. GISTs are mesenchymal tumors rarely arising from the caecum. Colon and rectum contribute to less than 5% of all GISTs and only about 4–5% of all GISTs with typical morphological features are negative for CD117 [3,4]. As these tumors grow in an extraluminal fashion, intestinal obstructions and intussusception are uncommon [5].

In the literature, very few cases of bowel intussusceptions from a stromal tumor in adults have been described. Here, we present a case of an adult female with ileocolic intussusception due to underlying CD117 negative cecal GIST. The coexistence of these pathologically distinct and rare entities has seldom been reported and represents a unique finding. This case has been reported in line with SCARE criteria [6].

## 2 Case presentation

A 30-year regularly menstruating non-alcoholic and non-smoker, previously healthy female presented to our center with a complaint of insidious onset, severe, colicky, and non-radiating right lower abdominal pain for 10 days along with multiple episodes of vomiting containing ingested food particles. However, there was no fever, hematochezia/melena, weight loss, or trauma to the abdomen before the onset of the symptom. There was no surgical history or any family history of malignancy.

On examination, she was afebrile with a BP of 100/80 mmHg, pulse rate of 80 beats per minute, respiratory rate of 15 breaths per minute, and SpO_2_ of 97% in the room air. On abdominal examination, the lower abdomen was soft, tender, distended with increased bowel sound without any sign of peritonitis. Examination of all other systems was grossly normal.

**Fig. 1:**
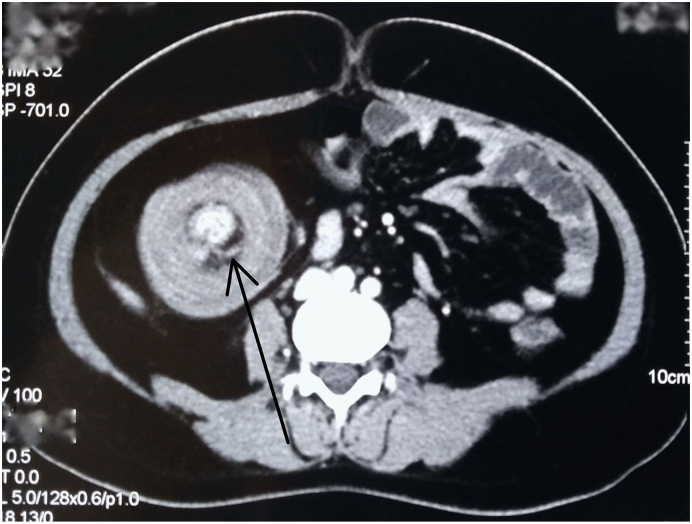
CECT abdomen (axial section) showing telescoping of the ileal segment into ascending colon with target sign (arrow).

Blood counts were within normal limits with Hb of 11.1 g%, total count of 12,000 and platelets of 351,000, serum sodium of 134 mEq/L, serum potassium of 3.6 mEq/L, RBS of 4.1 mmol/L (3.1–7.8), urea of 3.7 mmol/L (1.6–7.0), and creatinine of 58 μmol/L (40–110). Plain abdominal radiograph revealed dilated small bowel loops and multiple air-fluid levels suggesting small bowel obstruction. A contrast-enhanced CT scan of the abdomen showed telescoping of ileal segments into ascending colon (Fig. 1).

**Fig. 2:**
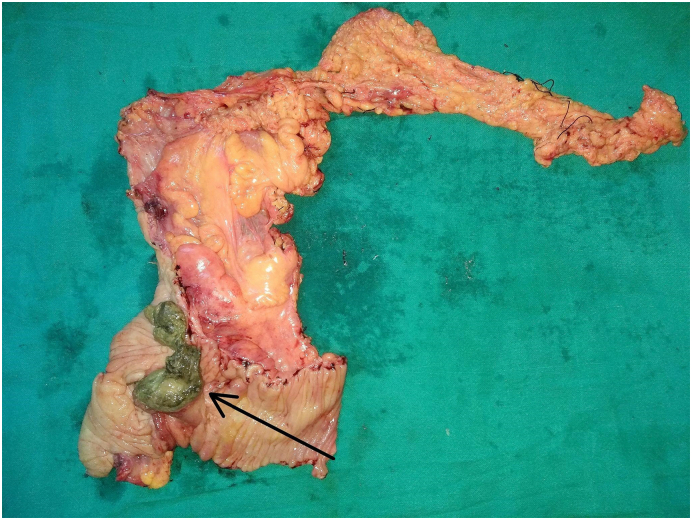
Right hemicolectomy specimen showing polypoidal mass originating from the cecum (arrow).

With the suspicion of intussusception causing intestinal obstruction, she was resuscitated with intravenous fluids along with nasogastric decompression and intravenous antibiotics. Emergency laparotomy was done and on exploring the abdomen, ileal loops invaginating into ascending colon with proximal dilated bowel loops were revealed. After the reduction of the intussusception, a hard mass was felt at the cecum. With suspicion of a malignant lesion, a standard right hemicolectomy was performed by the experienced team of gastrointestinal surgeons of Tribhuvan University Teaching Hospital. On opening the specimen, a pedunculated polypoid hard mass of size 9 cm × 5 cm × 3 cm was seen arising from the posteromedial wall of the cecum with separated visualization of the appendix (Fig. 2).

**Fig. 3:**
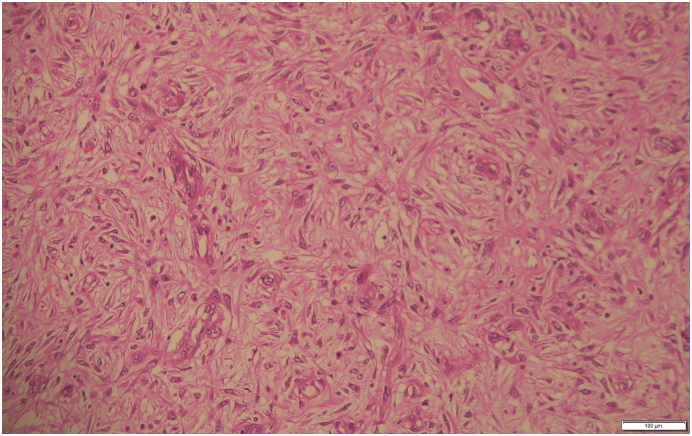
Spindle-shaped tumor cells having eosinophilic cytoplasm and elongated nuclei with collagenized stroma (H&E ×400).

**Fig. 4:**
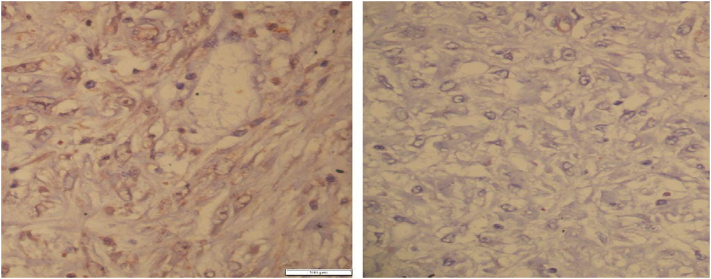
These spindle-shaped tumor cells show moderately intense cytoplasmic reactivity to DOG-1 immunostain (left, IHC DOG-1 ×400) and are negative for CD117 immunostain (right, IHC CD117(c-KIT) ×400).

Histopathological examination of the excised mass showed spindle-shaped cells arising from the muscularis propria and arranged in fascicles with oval to elongated nuclei having mild nuclear polymorphism and vesicular chromatin along with dense infiltration of lymphocytes in the intervening collagenized stroma (Fig. 3). The mitotic figure was infrequent (0 per 50 HPF). DOG-1 immunostain showed moderately intense cytoplasmic positivity in the tumor cells confirming the diagnosis of GIST, spindle cell type, moderate risk category (Fig. 4) However, CD117 (c-KIT) immunostain was negative.

The post-operative period was uneventful and the patient recovered well. The patient was started on imatinib considering the large size of the tumor with a moderate-risk category. The patient is doing well and remains disease-free at 6 months of follow-up, and is happy with the treatment she is receiving.

## 3 Discussion

GISTs are rare gastrointestinal tumors that arise from interstitial cells of Cajal or stem precursors cell and commonly seen among male above 40 years of age [7,8]. The patients of GIST usually present with symptoms of bleeding per rectum and other partial or complete gastric outlet obstruction complaints like abdominal pain, nausea, vomiting, and early satiety depending on the location of the tumor. Intussusception secondary to GIST is a rare complication and cecal GIST causing ileocolic intussusception is even rarer [9]. Intussusception in adults presents with vague symptoms which is the main cause for misdiagnosis or delayed diagnosis. Our patient presented with lower abdominal pain, vomiting, and abdominal distension. Cecum or appendix is the common location for the lead point of enterocolic intussusception through which a GIST propels through the ileum into the cecum [10]. This might be the probable cause of intussusception in our case too. An accurate diagnosis of intussusceptions should include a good history, a thorough physical examination, radiography, CT, MRI, and enteroclysis, or even an endoscopic ultrasound or capsule endoscopy. As the findings of suspected GISTs on ultrasound are indistinct, the best imaging modality for evaluation in patients with non-specific symptoms is a contrast-enhanced CT scan. This helps to know the extent of the tumor and the presence of metastatic spread and also helpful in the diagnosis of intestinal obstruction [11]. However, the preoperative diagnosis is often difficult owing to the non-specific presentation of the tumor.

Most of the surgeons opt for surgical procedures among adults with intussusception because of its greater incidence with structural anomalies and malignancy [12]. The operation of choice among many surgeons is en bloc resection with a negative margin because ileocolonic and colon-colonic lesions have a greater incidence of malignancy [13]. The metastasis and risk of local recurrence of GISTs are decreased with complete resection of the localized lesion [14]. Histopathological examination and immunohistochemistry studies help in the accurate diagnosis of GISTs. CD117 is a specific and sensitive marker in the diagnosis of GISTs [15]. However, with the discovery of DOG-1, it is considered a more sensitive and specific marker in the diagnosis of GIST on cytologic specimens and is recommended in negative c-KIT [16]. The immunohistochemistry of the excised mass in our case was positive for DOG1 and negative for CD117, confirming the diagnosis of cecal GIST.

Tumor size and mitotic index help in determining the malignancy potential of GIST. Intestinal GISTs with size >5 cm have moderate risk while tumors with 5 mitoses per 50 high power field mitotic activity have high risk [17]. The mitotic figures in our case are 0 per 50 HPF but the size was less than 10 cm indicating its moderate risk of metastasis.

C-kit-positive GISTs are treated with surgery and/or imatinib. For patients with intermediate and high-risk GIST, adjuvant imatinib therapy after surgery is required which reduces the recurrence rate and improves survival [18]. As there is possibility of KIT or PDGFRA mutation in c-kit-negative GISTs, complete resection and adjuvant imatinib should be considered [19]. However, there is possibility of developing imatinib resistance in c-kit-negative GISTs due to *PDGFRA* D842 mutations [20]. Our patient is receiving adjuvant imatinib considering the size of the tumor and risk of recurrence. Even after complete surgical resection, recurrence of GIST can occur, thus the patient needs regular follow-up and investigations for local and distant recurrences.

## 4 Conclusion

Cecal GIST presenting in the form of adult ileocolic intussusception is rare and poses adiagnostic challenge for a surgeon because of its non-specific presentation. This entity has to be considered in adults presenting with intussusception. Surgical resection is the mainstay of the treatment.

## Declaration of competing interest

None.
